# Prevalence of *Clostridioides difficile* in Canine Feces and Its Association with Intestinal Dysbiosis

**DOI:** 10.3390/ani13152441

**Published:** 2023-07-28

**Authors:** Melanie Werner, Patricia Eri Ishii, Rachel Pilla, Jonathan A. Lidbury, Joerg M. Steiner, Kathrin Busch-Hahn, Stefan Unterer, Jan S. Suchodolski

**Affiliations:** 1 Clinic for Small Animal Internal Medicine, Vetsuisse Faculty, 8057 Zurich, Switzerland; 2 Gastrointestinal Laboratory, Department of Small Animal Clinical Sciences, School of Veterinary Medicine and Biomedical Sciences, Texas A&M University, College Station, TX 4474, USA; 3 Clinic of Small Animal Internal Medicine, Centre for Clinical Veterinary Medicine, Ludwig Maximilians University, 80539 Munich, Germany

**Keywords:** microbiota, microbiome, enteropathogens, dog, Clostridium

## Abstract

**Simple Summary:**

The impact of *Clostridioides (C.) difficile* on gut diseases in dogs is a subject of debate. In people, an imbalance in bile acids in the gut is often linked to the presence of *C. difficile*. This study examined the relationship between an imbalance in the gut bacteria (altered dysbiosis index and decreased *C. hiranonis*) and the presence of *C. difficile* in dogs. We looked at the following four different groups: dogs with digestive issues (submitted samples and those with long-term or short-term diarrhea) and healthy dogs. Our results showed that dogs with *C. difficile* often had a greater imbalance in gut bacteria and less of another type of bacteria (*C. hiranonis*). Importantly, regardless of whether dogs were carriers of *C. difficile,* this did not influence how well dogs responded to treatments for digestive problems. This suggests that, in dogs, the presence of *C. difficile* does not require special treatment. In short, while *C. difficile* is associated with microbiota dysbiosis and lower levels of *C. hiranonis* in dogs, its presence does not require changes in routine treatment.

**Abstract:**

The role of *Clostridioides (C.) difficile* as an enteropathogen in dogs is controversial. In humans, intestinal bile acid-dysmetabolism is associated with *C. difficile* prevalence. The relationship between fecal qPCR-based dysbiosis index (DI) and especially the abundance of bile acid-converting *Clostridium hiranonis* with the presence of *C. difficile* in dogs was explored across the following 4 cohorts: 358 fecal samples submitted for routine diagnostic work-up, 33 dogs with chronic enteropathy, 14 dogs with acute diarrhea, and 116 healthy dogs. Dogs that tested positive for *C. difficile* had significantly higher DI (median, 4.4 (range from 0.4 to 8.6)) and lower *C. hiranonis* (median, 0.1 (range from 0.0 to 7.5) logDNA/g) than dogs that tested negative for *C. difficile* (median DI, −1 (range from −7.2 to 8.9); median *C. hiranonis* abundance, 6.2 (range from 0.1 to 7.5) logDNA/g; *p* < 0.0001, respectively). In 33 dogs with CE and 14 dogs with acute diarrhea, the treatment response did not differ between *C. difficile*-positive and -negative dogs. In the group of clinically healthy dogs, 9/116 tested positive for *C. difficile*, and 6/9 of these had also an abnormal DI. In conclusion, *C. difficile* is strongly linked to intestinal dysbiosis and lower *C. hiranonis* levels in dogs, but its presence does not necessitate targeted treatment.

## 1. Introduction

*Clostridioides (C.) difficile* is an anaerobic, toxin-producing bacillus present in the intestinal tract of various animal species and humans [1,2]. The role of intestinal *C. difficile* in dogs as a potential pathogen is controversial. In humans, its clinical significance is better understood. Antibiotic administration and inflammatory bowel disease leading to the disruption of the normal microbiota are risk factors for infection [2,3,4,5]. Moreover, *C. difficile* is one of the most significant nosocomial infectious organisms in humans, as carrying *C. difficile* toxins A/B can provoke clinical signs, including severe diarrhea [3,4]. However, carrying *C. difficile* alone does not cause clinical signs, as approximately 5% of healthy humans are colonized by *C. difficile* [2]. The virulence of *C. difficile* is based on the toxins A and B, which can both form microtubule-based protrusions on epithelial cells that can result in damage to the epithelial lining [6,7]. The major protective barrier against *C. difficile* infection is the functioning intestinal microbiota [8,9]. After the disruption of the normal intestinal microbiota, *C. difficile* can dominate the large intestine in humans [10]. Moreover, in humans, bacterial species (e.g., *C. scindens*) that are part of the normal microbiota and play an essential role in the conversion of primary to secondary bile acid competitively inhibit the growth of *C. difficile* [11,12,13,14,15,16]. A negative association between the occurrence of *C. difficile* and bile acid-converting bacteria has been reported in humans [14]. This is either due to a direct antimicrobial effect of secondary bile acids and/or competition between bile acid-converting bacteria and *C. difficile* for nutrients (e.g., proline and glycine) [16]. Although several studies have shown that in dogs, *C. hiranonis* is the primary bacterium involved in bile acid conversion, few data exist describing the association between the presence of *C. difficile* and decreased abundance of *C. hiranonis* in the feces of dogs [17,18,19,20]. 

The dysbiosis index (DI) is a standardized test that allows the assessment of microbiome shifts. A recent meta-analysis of 27 studies has demonstrated that the DI, which is calculated based on the relative abundance of core bacteria such as the bile acid-converting *C. hiranonis*, is a useful biomarker of intestinal functionality. *C. hiranonis* is often decreased in dogs with chronic enteropathies and dogs that have received broad-spectrum antibiotics, such as metronidazole and tylosin [21,22,23,24]. Many commercial fecal enteropathogen panels assess the presence of *C. difficile* in dogs presenting with diverse gastrointestinal disorders, such as chronic enteropathies (defined as chronic gastrointestinal signs > 3-week duration and exclusion of extra-intestinal causes of the disease) or acute idiopathic diarrhea (diarrhea for less than 1-week duration without known etiology) without evaluating the microbiome composition. Moreover, many clinicians still treat *C. difficile* carriers with antimicrobials without proper indication. Consequently, a proper understanding of the clinical importance of *C. difficile* in dogs and its potential association with an intestinal dysbiosis is important to better guide treatment decisions.

Thus, the aim of this retrospective study was to investigate the association between *C. difficile*, *C. hiranonis*, and the DI in a large number of fecal samples of dogs submitted to a commercial laboratory. The second objective of this study was to assess the association between *C. difficile* and clinical signs in fecal samples obtained from three distinct cohorts of dogs, with well-characterized clinical presentation and clinical outcomes and long-term follow-up. These three cohorts comprised dogs with chronic enteropathy (CE), idiopathic uncomplicated acute diarrhea, and clinically healthy dogs. 

## 2. Materials and Methods

### 2.1. Samples

The study was a retrospective cohort study. The following four different cohorts of dogs were studied: Cohort 1 comprised a large number of fecal samples from dogs submitted to a commercial laboratory with unknown clinical presentation (*n* = 358). To assess association between presence of *C. difficile* and clinical outcome, the following 3 additional cohorts with well-known clinical work-up and follow-up were evaluated: Cohort 2 consisted of 33 dogs with chronic enteropathy, Cohort 3 included 14 dogs with acute idiopathic diarrhea, and cohort 4 consisted of 116 healthy dogs. 

For Cohort 1, results from canine fecal samples submitted to the Gastrointestinal Laboratory of the School of Veterinary Medicine and Biochemical Science of the Texas A&M University (between 2016 and 2021) were reviewed. This is a commercial laboratory that determines the dysbiosis index at the request of various veterinarians. Canine fecal samples in which measurements of both the DI and qPCR for *C. difficile* were available were used for statistical analysis. Moreover, the database was screened for *C. difficile*-positive samples in which concurrent *C. difficile* toxins A/B ELISA results were available. The clinical history, reason for submission, and outcome data of Cohort 1 were unknown. 

In Cohort 2, dogs with chronic enteropathy with available clinical data and long-term follow-up were included. Dogs of either sex, neuter status, bodyweight, and at least 1 year of age with chronic gastrointestinal signs and chronic diarrhea for at least 3 weeks were included. Exclusion criteria were pretreatment with antimicrobials and corticosteroids within the last 3 months. Standardized work-up of these dogs included CBC (complete blood count; all dogs), serum chemistry profile (29/33), fecal flotation (32/33), Giardia antigen ELISA (31/33) and measurement of cobalamin (29/33), folate (29/33), fasted serum bile acids (3/33), TLI (trypsin-like immunoreactivity; 17/33), cPLI (canine specific pancreatic lipase; 17/33), basal cortisol/ACTH-stimulation test (14/33) and an abdominal ultrasound (13/33) depending on what the responsible clinician has deemed necessary to diagnose the dog with a chronic enteropathy. The dogs were then subgrouped according to their response to treatment as food responsive (trial with a hydrolyzed or exclusion diet for at least 2 weeks), immunosuppressive responsive (prednisolone < 2 mg/kg PO q24 h), or antibiotic responsive (metronidazole or tylosin). Response to therapy was evaluated in all dogs during work-up at regular in-person visits to the veterinarian. In all dogs, additionally long-term response to treatment was assessed by telephone interview 1 year after initial presentation (CIBDAI-Index (Canine Inflammatory Bowel Disease Activity Index), treatment response (yes/no), kind of treatment, diet). Since discrimination of patients with regards to response to one of the aforementioned therapeutic strategies is neither possible with laboratory diagnostic nor histopathological tests, the response to therapy was evaluated purely clinically. The dysbiosis index was measured before any initiation of treatments (e.g., antimicrobials). Moreover, the CIBDAI score was determined and compared between the two timepoints (at first presentation and at the follow-up). 

Cohort 3 comprised leftover fecal samples of dogs with acute idiopathic diarrhea that were described in a previous study, but *C. difficile* was not reported then [25]. The aim of the previous study was to assess whether amoxicillin-clavulanic acid has a clinical benefit in dogs with acute uncomplicated diarrhea. Inclusion criteria were the following: dogs of either sex with acute non-hemorrhagic diarrhea for less than 3 days between 5 and 40 kg bodyweight, and of at least 9 months of age were included into this cohort. Exclusion criteria were pretreatment with an antimicrobial within 30 days or treatment with an anti-inflammatory drug within 7 days before presentation, blood in feces, any signs of systemic inflammation, severe illness, or significant dehydration prompting hospitalization. The dogs were presented to a university clinic and privately owned hospitals. The CADS (canine acute diarrhea severity) score was determined in all dogs. Moreover, in all dogs a CBC and serum chemistry profile was performed to exclude dogs with extraintestinal causes of the disease. Endoparasites were excluded by fecal flotation. All dogs received symptomatic treatment (highly digestive diet, antiemetic maropitant, and analgesic metamizole), and half of the dogs (7/14) additionally received amoxicillin clavulanic acid (10–20 mg/kg PO q12h) after obtaining the fecal sample.

Cohort 4 consisted of clinically healthy dogs. The dogs had no clinically relevant history or findings on physical examination. Exclusion criteria were gastrointestinal signs or administration of antimicrobials or probiotics during the last 4 weeks before taking the fecal sample. One fecal sample per dog was taken extracorporeally.

### 2.2. Microbiota Analysis

#### 2.2.1. DNA Extraction

All fecal samples (cohorts 1, 2, 3, and 4) were processed immediately after shipment to the laboratory or were preserved at −80 °C. DNA was extracted from an aliquot of 100 mg of each fecal sample using a MoBio Power soil DNA isolation kit (MoBio Laboratories, Carlsbad, CA, USA) according to the manufacturer’s instructions. The bead-beating step was performed on a homogenizer (FastPrep-24; MP Biomedicals, Santa Ana, CA, USA) at a speed of 4 m/s for 60 s. The fecal DNA was frozen at −80 °C until further analysis.

#### 2.2.2. Quantitative PCR (qPCR)

The abundance of total bacteria and seven bacterial taxa (i.e., *Faecalibacterium* spp., *Turicibacter* spp., *Streptococcus* spp., *E. coli*, *Blautia* spp., *Fusobacterium* spp., and *C. hiranonis*), which had been identified as being altered in dogs with gastrointestinal disease in previous studies, were quantified by specific qPCR assays and used to calculate the DI as described in various previous studies [21,22,23,26,27]. Moreover, *C. difficile* PCR was performed as previously described [28]. Shortly, the qPCR assay for detecting the cpe gene and cdt b gene in feces used a 10 μL total volume. The mastermix included 5 μL of SsoFast Probes supermix, 2.35 μL of water, 0.25 μL of each primer (250 nM final concentration), 0.15 μL of the probe (150 nM final concentration), and 2 μL of DNA. The qPCR cycling conditions comprised an initial incubation at 95 °C for 2 min, followed by 40 cycles of denaturation at 95 °C for 5 s and annealing for 10 s at the optimized annealing temperature. The qPCR assays were conducted using a commercial real-time PCR thermal cycler (CFX96 Real-Time PCR Detection System, Biorad Laboratories, Hercules, Kalifornien, USA), with all samples run in duplicate. The oligonucleotide sequences of the primers and probes and the annealing temperatures are documented in Table S1 [29,30,31,32,33,34,35]. A DI > 2 was defined as significant dysbiosis and values between 0 and 2 as mild dysbiosis [23].

#### 2.2.3. Toxin Immunoassay

*C. difficile* toxins A/B were detected using a validated commercially available ELISA kit (*C. DIFFICILE* TOX A/B II™ TechLab, Itasca, IL, USA), and that was also used previously in dogs [36,37]. The test was performed according to the manufacturer’s instructions. Feces were diluted in 200 µL of diluent and mixed for 10 s. An aliquot of 100 µL of the diluted sample was then transferred to the microassay plate containing the detecting polyclonal antibody against toxins A and B. The ELISA reaction was then examined spectrophotometrically using a commercial multimode microplate reader (Synergy 2 Multi-Mode Microplate Reader, BioTek, Winooski, VT, USA) at a wavelength of 450 nm. Samples were considered positive when an optical density >0.120 was reached, whereas an optical density <0.120 was considered negative. 

### 2.3. Statistical Analyses

Statistical analyses were performed with GraphPad Prism c7.0, (GraphPad Software, San Diego, CA, USA). Testing for normality was performed using the D’Agonisto-Pearson omnibus normality test. The association between categorical variables (i.e., sex, treatment responses) with *C. difficile*-positive and negative dogs was calculated using Fisher’s exact test. In addition, differences in continuous data (i.e., age, weight, DI, abundances of *C. difficile* and *C. hiranonis*, CADS and CIBDAI score) between *C. difficile*-positive and -negative dogs were evaluated using the unpaired *t*-test or the Mann–Whitney U test, depending on normality. Statistical significance was set at *p* < 0.05.

## 3. Results

### 3.1. Cohort 1: Sent in for Commercial Purposes by Various Veterinarians

For 358 dogs, concurrent results for the DI (including the abundance of *C. hiranonis*) and the *C. difficile* 16S rRNA gene were available. In 15/358 dogs, concurrent measurements of *C. difficile* by qPCR and the *C. difficile* toxins A/B by ELISA were available.

#### 3.1.1. Association between *C. difficile*/Dysbiosis Index and Toxin Measurement

*C. difficile* was detected in 130 of the 358 samples (36%). Samples that were *C. difficile*-positive showed DI > 0 in 115 of the 130 (89%) cases. Among the samples that tested negative for *C. difficile* (*n* = 228), 102 showed a DI > 0 (45%), whereas 126 samples had a DI < 0 (55%). The odds of carrying *C. difficile* were 9.5 (95% CI [8.9; 10.1]) times higher in dogs with an abnormal DI than they were in dogs with a normal DI. 

**Figure 1:**
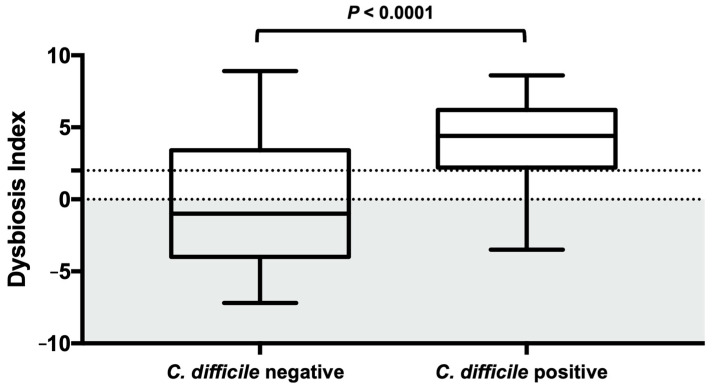
Dysbiosis index in *C. difficile*-negative and -positive dog fecal samples. The reference interval is shaded in grey. Dogs that tested positive for *C. difficile* had a significantly higher DI than did dogs that tested negative for *C. difficile*. Box and whisker plot showing minimum, maximum, median, and interquartile range are shown. Grey section represents the reference interval, section between the dotted lines represent grey zone auf the Dysbiosis Index.

**Figure 2:**
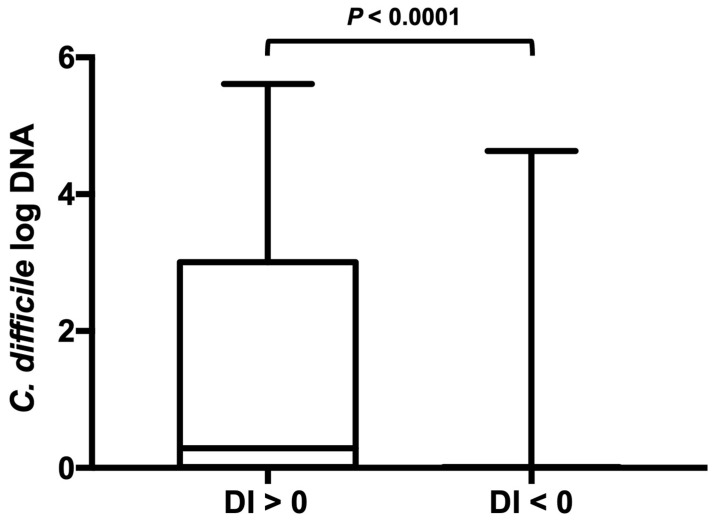
*C. difficile* abundance in dog fecal samples with an abnormal or normal dysbiosis index. *C. difficile* was found at greater abundance in dogs with an elevated DI > 0 compared with dogs with a low DI. Minimum, maximum, and median are shown. Box and whisker plot showing minimum, maximum, median, and interquartile range are shown. Since the minimum, 25% percentile, median, and 75% percentile are equal in the right-sided plot they are not displayed as separate lines.

Dogs that tested positive for *C. difficile* had a significantly higher DI (median, 4.4 (range from −3.5 to 8.6)) than did dogs that tested negative for *C. difficile* (median, −1 (range from −7.2 to 8.9) (*p* < 0.0001) (Figure 1). Moreover, differences were observed in the groups’ abundance of detected *C. difficile*. *C. difficile* was found in greater abundance in dogs with a DI > 0 compared to dogs with a DI < 0 (*p* < 0.0001) (Figure 2).

All 15 out of 358 dogs, for which concurrent measurements of *C. difficile* and toxin A/B ELISA results were available, tested negative for toxins A/B by ELISA.

#### 3.1.2. Association between *C. difficile* and *C. hiranonis*

**Figure 3:**
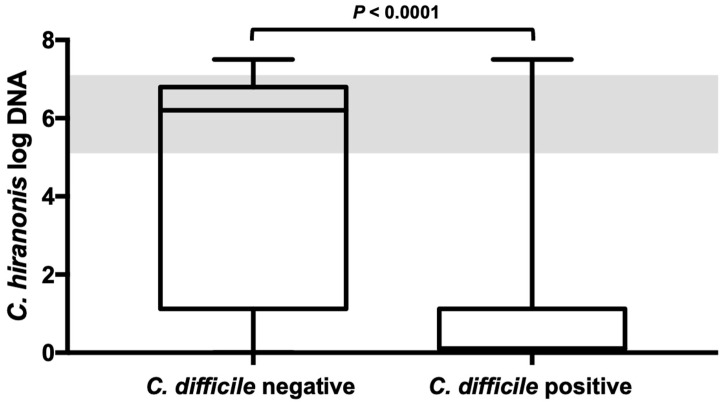
*C. hiranonis* abundance in dog fecal samples with and without *C. difficile*. Dogs tested positive for *C. difficile* had significantly lower abundances (logDNA/g) of *C. hiranonis* than did dogs that tested negative for *C. difficile*. The shaded area represents the reference interval. Minimum, maximum, and median are shown. Box and whisker plot showing minimum, maximum, median, and interquartile range are shown. Since the 25% percentile and median are equal in the right-sided plot they are not displayed as separate lines. Grey colored area represents the reference interval.

Samples that were *C. difficile*-positive showed a decreased abundance below the reference range of *C. hiranonis* in 113 out of the 130 (87%) cases. Among the samples that tested negative for *C. difficile* (*n* = 226), 70 showed a decreased abundance of *C. hiranonis* (31%), whereas 156 had a normal *C. hiranonis* abundance (69%). The odds for carrying *C. difficile* were 14.8 (95% CI [14.2; 15.4]) times higher in dogs with decreased *C. hiranonis* abundance than they were in dogs with a normal quantity of *C. hiranonis* within the reference interval. Dogs that tested positive for *C. difficile* had significantly lower abundance (logDNA/g) of *C. hiranonis* (median, 0.1 (range from 0.01 to 7.5)) than did dogs that tested negative for *C. difficile* (median, 6.2 (range from 0.01 to 7.5)) (*p* < 0.0001) (Figure 3). Moreover, *C. difficile* was found in greater abundance in dogs with abnormally decreased *C. hiranonis* abundance compared to dogs with normal *C. hiranonis* (*p* < 0.0001).

### 3.2. Cohort 2: Cohort of Dogs with Chronic Enteropathy

History and treatment responses were available for 33 dogs with chronic enteropathy, with their demographic data summarized in Table 1. The median CIBDAI Index prior to treatment was 7 (range 2–14), and at reassessment after initiation of treatment, the median was 1 (range 0–8). Signalment and disease activity scores (CIBDAI) at first presentation were not significantly different between dogs that tested positive and negative for *C. difficile*. In this cohort of dogs, a DI > 0 was found in 19 (58%), hypocobalaminemia in 4 (12%), and hypofolatemia in 5 (15%) of the 33 dogs at the timepoint of the first presentation. The treatment with which the clinical signs of the dog disappeared was used for the classification of treatment response. Non-responders refer to dogs that did not respond to dietary changes, steroids, or antibiotics. The final classification can be found in Table 1. There was no significant difference in the treatment response between *C. difficile*-positive and *C. difficile*-negative dogs. 

### 3.3. Cohort 3: Dogs with Acute Idiopathic Diarrhea

In total, 14 dogs with acute diarrhea (median age 7.5 years, range 3–11 years; 6 females, 8 males; Table 2) of different breeds were included. Of these 14 dogs, only 1 dog (7%) tested positive for *C. difficile*. This dog showed a concurrent abnormal DI of 5.4 and had decreased *C. hiranonis*. In the remaining 13/14 dogs with acute diarrhea, a DI > 0 was found in 4/13 (30%) and a DI > 2 in one dog. All dogs recovered (based on an assessment of the CADS Index) with symptomatic treatment (7/14) or symptomatic treatment and antimicrobial treatment (amoxicillin clavulanic acid; 7/14) within 10 days after presentation. The one dog that tested positive for *C. difficile* received only symptomatic treatment without any additional antimicrobial drugs. 

### 3.4. Cohort 4: Healthy Dogs

In total, 116 clinically healthy dogs (Table 3) were included in this cohort, consisting of 28 mixed-breed dogs and 88 different pure-breed dogs. The median age was 3.5 years (ranging from 6 months to 13 years), and the median bodyweight was 14.9 kg (ranging from 1.9 to 57 kg). Of these, 9/116 (8%) dogs tested positive for *C. difficile*, from which 6/9 (67%) showed a concurrent abnormal DI and 5/9 (56%) abnormal abundance of *C. hiranonis*. Samples that tested positive for *C. difficile* and had *C. hiranonis* levels within the reference range exhibited a significantly lower abundance (logDNA/g) of *C. difficile* (median, 0.16 (ranging from 0.06 to 0.31) compared to samples with *C. hiranonis* levels below the reference range (median, 4.13 (range from 2.06 to 4.59); *p* = 0.0003). Of the remaining dogs, which tested negative for *C. difficile*, only 7/116 (4%) showed an abnormal DI, with 4/7 having a concurrent low abundance of *C. hiranonis*.

## 4. Discussion

This study describes the association between the presence of *C. difficile*, an increased dysbiosis index, and decreased abundance of the bile acid-converting bacterium *C. hiranonis* in canine feces. In addition, it evaluated the clinical data in a cohort of dogs with CE, acute diarrhea, and clinically healthy controls based on their carrier status for *C. difficile*. This study found a strong association between the presence of *C. difficile*, the presence of dysbiosis, and an abnormally low abundance of *C. hiranonis*. Thus, if a positive result for *C. difficile* is obtained, further consideration should be given to the presence of intestinal dysbiosis. 

Profound dysbiosis was detectable in previous studies in dogs with chronic enteropathies and in dogs previously treated with antimicrobials (especially metronidazole or tylosin), whereas factors such as age, diet, and environment, only lead to minor changes in the microbiome composition, mostly within the reference intervals of the dysbiosis index [22,23,24,35]. Changes in the intestinal microbiota can cause functional changes, such as decreased short-chain fatty acid levels and abnormal bile acid metabolism [17,26,30]. The latter is considered an important contributing factor to antimicrobial-induced infections with *C. difficile* in humans. A balanced intestinal environment and physiologic bile acid metabolism are important for preventing the overgrowth of harmful bacteria, such as *C. difficile,* as shown in humans [16,38,39]. 

Bacteria that inhibit *C. difficile* are the previously mentioned bile acid-converting bacteria [38]. In humans, *C. scindens* plays the most important role in this context, as a negative association between *C. scindens* and intestinal bile acid dysmetabolism and, consequently, the presence of *C. difficile* is well established [14]. *C. hiranonis* is the essential bile acid converter in dogs [18]. *C. hiranonis* is often decreased in dogs with dysbiosis associated with chronic enteropathies or antibiotic administration but only rarely in acute diarrhea [21,22,25,40]. In the present study, in addition to the association between dysbiosis and *C. difficile* occurrence, we were able to demonstrate a negative association between the fecal abundance of *C. hiranonis* and *C. difficile* prevalence. However, it should be noted that a large number of samples in the present study also revealed a decreased abundance of *C. hiranonis* and dysbiosis without the presence of *C. difficile*. Thus, testing for *C. difficile* alone does not replace a more general microbiota analysis, such as the DI. 

The presence of *C. difficile* was relatively common in the samples evaluated. Furthermore, the proportion of *C. difficile*-positive samples detected in Cohort 1 is slightly higher in comparison to previous studies of its prevalence in healthy (14%) and diarrheic dogs (16%) [41,42]. However, in our healthy control cohort, the prevalence was only 8% and was associated with subclinical dysbiosis. In humans, the prevalence of *C. difficile* carrier status is estimated to be 5% in healthy individuals and is higher in individuals with an intestinal disorder that leads to a disruption of the microbiota and associated disruption of bile acid metabolism, such as inflammatory bowel disease [43,44]. The fact that most of the samples of Cohort 1 were sent in by veterinarians for a specific purpose would suggest that many of these dogs were diseased, which may explain the higher prevalence compared to the healthy dog group.

Cohorts of dogs with CE and acute diarrhea and known long-term treatment outcomes were examined more closely in the present study. The aim of evaluating these groups of dogs was to determine if the carrier status of *C. difficile* affects treatment response and if the outcome differs between *C. difficile*-positive and negative dogs. First, we examined whether the presence of C. difficile in the dogs with CE affected the clinical severity. For this purpose, the CIBDAI disease activity index was determined, which did not differ significantly between groups [45]. The causes of CE are mostly multifactorial and not fully understood. Nevertheless, in most cases, the classification of CE depends on the dog’s response to treatment [46]. In this study, dogs with CE, regardless of whether they tested positive or negative for *C. difficile,* showed a similar distribution in treatment response. Most of the dogs improved after dietary management, whereas fewer dogs required an anti-inflammatory treatment (e.g., steroid administration) or antimicrobial treatment. This result would suggest that the presence of *C. difficile* is strongly associated with intestinal dysbiosis in dogs with CE and that *C. difficile* does not need to be addressed separately, and that the standard therapeutic approach to CE remains unchanged. The treatment response in this study is in line with other retrospective evaluations in which most of the dogs were food responsive [46,47]. Moreover, one case report described five dogs with CE in which *C. difficile,* together with its toxins, was isolated [48]. All five dogs were unresponsive to treatment with metronidazole but responded clinically to dietary modulation [49]. Therefore, these data suggest that the presence of *C. difficile* in the feces of dogs with chronic diarrhea should not be seen as a primary enteropathogen and is not an indication for antibiotic treatment. An intestinal dysbiosis, together with decreased *C. hiranonis* and subsequent bile acid dysmetabolism occurs, commonly in dogs with CE. In our patient Cohort 2, serum folic acid and cobalamin concentrations were less frequently altered than the dysbiosis index, indicating that intestinal dysbiosis occurs commonly in CE. There is evidence that the intestinal microbiota plays an important role in chronic enteropathies in humans and dogs [17,49,50]. Therefore, further studies are warranted to evaluate whether there are, adjunct to standard therapy, additional benefits of microbiota restauration in dogs with chronic enteropathies with the help of, for example, probiotics and/or fecal microbiota transplantation [51,52,53].

In the group with acute idiopathic diarrhea, *C. difficile* was detected in only one dog at presentation. This dog showed concurrent abnormal DI combined with a low abundance of *C. hiranonis,* which can be seen transiently in a subset of dogs with acute diarrhea [25,40]. Importantly, this dog recovered with symptomatic treatment alone without any need for further antimicrobial therapy. Similar findings have also been reported in a previous study evaluating clinical outcomes in dogs with acute hemorrhagic diarrhea syndrome (AHDS), in which 18% of dogs were positive on qPCR for *C. difficile* toxin *cdt-b*, which was not significantly different from healthy dogs (13% positive) [30]. Only 1% tested positive for both the organism on PCR and the toxin by ELISA. There was also no association between clinical severity and outcome and *C. difficile* status. The authors concluded that *C. difficile* does not appear to play a causal role, at least in the majority of dogs with AHDS.

In human medicine, *C. difficile* plays a major role as a disease-causing pathogen. The inflammation-triggering factors are toxins A and B, produced by *C. difficile* (enterotoxin TcdA; cytotoxin TcdB). Since, not only the presence of *C. difficile* alone, but the presence of toxin-producing strains is relevant, 15 dogs that tested positive for *C. difficile* on qPCR were also tested for the presence of the toxin itself by ELISA. All of these were negative. This result further underlines the theory that *C. difficile* plays a minor clinical role in dogs in contrast to humans.

Our study had several limitations. The results from Cohort 1 were collected retrospectively from a database of veterinary diagnostic specimens. Thus, no information was available regarding the clinical signs and medical history. However, the major aim of this study was to evaluate the associations between the presence of *C. difficile*, intestinal dysbiosis, and the abundance of the bile acid-converting bacterium *C. hiranonis*. Therefore, the large number of samples that could be evaluated is a major strength of this study. In addition, the prevalence of *C. difficile* in cohorts 2 and 3 with known clinical response to therapy was similar to those obtained for the large Cohort 1. Further studies with a larger cohort of dogs that are prospectively evaluated for their clinical response over time, and multiple measurements of *C. difficile* over time, would be desirable. Another limitation of this study was that only 15 *C. difficile*-positive samples were tested for the presence of the toxin by ELISA. Nevertheless, one important result of this study was that none of the 15 dogs harboring *C. difficile* tested positive for the toxin; therefore, their clinical relevance, at least in this population of dogs, can be considered low. However, translation of these results to the general population of dogs with positive *C. difficile* requires further study, as there are some published case reports on dogs that tested positive for *C. difficile* and toxin A/B with concurrent clinical signs [54,55]. Lastly, less is known about the analytical sensitivity of the toxin A/B ELISA in dogs. A low sensitivity might have led to an underestimation of the number of dogs that carried toxin A/B. While internal laboratory data showed a strong positive correlation between the abundance of *C. difficile* by qPCR and a positive test result by ELISA, it is possible that the presence of the toxin may have been missed in those dogs carrying a very low abundance of *C. difficile*. 

## 5. Conclusions

This study investigated the link between the presence of *C. difficile*, abnormal dysbiosis index, and low levels of *C. hiranonis*, a bile acid-converting bacteria, in dog feces. It found that dogs with *C. difficile* often displayed an abnormal dysbiosis index and lower *C. hiranonis* levels. The presence of *C. difficile*, however, did not affect treatment response in dogs with chronic or acute diarrhea, suggesting *C. difficile* itself does not need separate treatment. 

## Figures and Tables

**Table 1:** Demographics and treatment response of 33 dogs with chronic enteropathy and known long-term clinical outcome data.

	*C. Difficile*-Negative (*n* = 25)	*C. Difficile*-Positive (*n* = 8)	*p*-Value
demographic data			
Age in years (median (range))	5 (0.5–12)	5 (1–10)	0.97
Male (n, %); female (n, %)	Male (13, 52%); female (12, 48%)	Male (5, 63%); female (3, 37%)	0.60
Weight (kg) (median)	10.2	15.5	0.72
CIBDAI (median (range))	7 (2–12)	7.5 (5–14)	0.68
treatment response in % of dogs (x/y)			
FRE	63% (17/25)	75% (6/8)	0.70
SRE	19% (5/25)	12.5% (1/8)	0.63
ARE	4% (1/25)	12.5% (1/8)	0.38
Non-responders	7% (2/25)	-	0.41
Total	100% (25/25)	100% (8/8)	

CIBDAI = canine inflammatory bowel disease activity index; FRE = food-responsive enteropathy; SRE = steroid-responsive enteropathy; ARE = antibiotic-responsive enteropathy.

**Table 2:** Demographics, dysbiosis index and *C. difficile* carriage of 14 dogs with acute idiopathic diarrhea.

	*C. difficile*-Negative (*n* = 13)	*C. difficile*-Positive (*n* = 1)
Age in years (median (range))	7 (3–11)	4
Male (n, %); female (n, %)	Male (7, 54%); female (6, 46%)	Male (1, 100%); female (0, 0%)
Weight (kg) (median)	12.0	15.0
Dysbiosis index > 0 (n (%))	4 (30%)	1 (100%)
Dysbiosis index < 0 (n (%))	9 (70%)	0 (0%)

**Table 3:** Demographics, dysbiosis index and *C. difficile* carriage of 116 healthy dogs.

	*C. difficile*-Negative (*n* = 107)	*C. difficile*-Positive (*n* = 9)
Age in years (median (range))	3.25 (0.5–13)	7.5 (5–12)
Weight (kg) (median)	13.3	23.5
Dysbiosis index > 0 (n (%))	7 (7%)	6 (66%)
Dysbiosis index < 0 (n (%))	100 (93%)	3 (33%)

## Data Availability

Data available on request due to restrictions eg privacy or ethical.

## Supplementary Materials

The following supporting information can be downloaded at: https://www.mdpi.com/article/10.3390/ani13152441/s1, Table S1: Oligonucleotides primers/probes used in this study.
