# Supplementary material for: Prevalence of Clostridioides difficile in Canine Feces and Its Association with Intestinal Dysbiosis
Source: Animals (Basel). 2023 Jul 28;13(15):2441. doi: 10.3390/ani13152441 (PMC10417777; doi:10.3390/ani13152441)
Supplement: Supplementary file 1 [file animals-13-02441-s001.zip › animals-2529556-supplementary.pdf]

Table S1. Oligonucleotides primers/probes used in this study.

qPCR primers/probe	Sequence (5’- 3’)	Target	Annealing (°C)	Reference
Forward	GAAGGCGGCCTACTGGGCAC	*Faecalibacterium*	60	[29]
Reverse	GTGCAGGCGAGTTGCAGCCT			
Forward	KGGGCTCAACMCMGTATTGCGT	Fusobacteria	51	[30]
Reverse	TCGCGTTAGCTTGGGCGCTG			
Forward	TCTGATGTGAAAGGCTGGGGCTTA	*Blautia*	56	[30]
Reverse	GGCTTAGCCACCCGACACCTA			
Forward	CCTACGGGAGGCAGCAGT	Universal Bacteria	59	[31]
Reverse	ATTACCGCGGCTGCTGG			
Forward	CAGACGGGGACAACGATTGGA	*Turicibacter*	63	[30]
Reverse	TACGCATCGTCGCCTTGGTA			
Forward	GTTAATACCTTTGCTCATTGA	*E. coli*	55	[32]
Reverse	ACCAGGGTATCTAATCCTGTT			
Forward	AGTAAGCTCCTGATACTGTCT	*C. hiranonis*	50	[33]
Reverse	AGGGAAAGAGGAGATTAGTCC			
Forward	TTATTTGAAAGGGGCAATTGCT	*Streptococcus*	54	[34]
Reverse	GTGAACTTTCCACTCTCACAC			
Forward	F-TTGAGCGATTTACTTCGGTAAAGA	*C difficile* 16S rRNA	61	[35]
Reverse	R-TGTACTGGCTCACCTTTGATATTCA			
Probe	P-CCACGCGTTACTCACCCGTCCG			
